# Neuroanatomically informed psychotherapy after mild brain injury: a single-case report of clinical improvement

**DOI:** 10.3389/fnhum.2026.1738846

**Published:** 2026-01-30

**Authors:** Rosario Bordón Guerra, Wenceslao Peñate Castro, Eilin Ferreiro Díaz-Vélis, Coralia Sosa Pérez, José Luis Hernández Fleta, Jesús Morera Molina

**Affiliations:** 1 Department of Psychiatry, Hospital Universitario de Gran Canaria Doctor Negrín, Las Palmas de Gran Canaria, Spain; 2 Department of Medical and Surgical Sciences, Psychiatry and Medical Psychology Knowledge Area, University of Las Palmas de Gran Canaria, Las Palmas de Gran Canaria, Spain; 3 Department of Clinical Psychology, Psychobiology and Methodology, Faculty of Psychology, University of La Laguna, San Cristóbal de La Laguna, Spain; 4 Department of Neurosurgery, Hospital Universitario de Gran Canaria Doctor Negrín, Las Palmas de Gran Canaria, Spain

**Keywords:** acquired brain injury, diffusion tensor imaging, functional compatibility, neuropsychology, psychotherapy, single-case study, tractography

## Abstract

Psychotherapeutic interventions following acquired brain injury often underestimate the influence of neural connectivity on emotional and cognitive recovery, contributing to treatment response variability. We present a single-case report of an adult with mild acquired brain injury, in whom diffusion tensor imaging (tractography) and neuropsychological assessment were integrated to guide individualized clinical planning. Baseline tractography revealed globally preserved white-matter organization with rightward asymmetries in the uncinate and superior longitudinal fasciculi–pathways involved in cognitive–emotional integration. This specific neuroanatomical profile informed a psychotherapeutic plan that prioritized attentional and experiential strategies, being functionally compatible with the preserved networks, over those requiring complex verbal-analytic processing. After 1 year of intervention (approximately 40 sessions), reassessment documented significant functional improvements, quantified by a reduction in anxiety to a non-clinical range (HADS-A: 11 → 6) and a normalization in cognitive flexibility (TMT-B: 190 → 128 s), without observable structural change. These findings validate the hypothesis that therapeutic change may arise from functional optimization within preserved networks rather than anatomical reorganization. This report proposes the functional compatibility framework as a transferable and theoretically grounded model for precision psychotherapy, bridging the gap between neuroscience and clinical practice.

## 1 Introduction

Psychotherapy has established itself as an effective means of promoting psychological change, yet variability in patient response remains substantial (Cammisuli and Castelnuovo, 2023; Cuijpers et al., 2019). However, even under structured conditions and with experienced therapists, considerable variability in patient response persists. This heterogeneity cannot be explained solely by differences among theoretical models or by common factors; it also depends on how well the chosen interventions fit the person’s actual neurocognitive capacities and functional context (Wampold and Imel, 2015). From this perspective, we introduce the construct of functional compatibility, defined as the degree of consistency between the cognitive–emotional demands of a psychotherapeutic technique and the neurofunctional resources available to the patient. This notion aligns with recent developments in precision psychotherapy and person–treatment fit (Lutz et al., 2021; Norcross and Lambert, 2019), while extending them by explicitly incorporating neurofunctional indicators as a criterion for clinical planning (Etkin and Mathalon, 2024; Menon, 2023; Nozais et al., 2023).

From a neuroscientific standpoint, the organization of white-matter tracts–particularly the uncinate fasciculus (UF), superior longitudinal fasciculus (SLF), and cingulum–have been linked to emotional regulation, empathy, and cognitive flexibility (Anderson et al., 2023; Bubb et al., 2018; Liu et al., 2024; Nono et al., 2024; Toller et al., 2023). Such evidence suggests that psychotherapeutic effectiveness may depend, in part, on how interventions mobilize preserved or compensatory networks rather than those compromised by injury. In mild acquired brain injury (ABI), subtle disruptions in cognitive–emotional integration may occur without marked neuropsychological deficits, leading to mismatches between therapeutic demands and functional viability. Case-based neuropsychological research has shown that individualized interventions guided by neuroanatomical or connectivity findings can yield meaningful behavioral and emotional improvements (Neymeyer et al., 2025).

This need for a neuro-informed approach is especially critical in cases of mild ABI, where subtle disruptions in white-matter integrity can lead to significant psychosocial disability. We report the case of an adult who presented with persistent emotional dysregulation, attentional instability, and difficulties in social interactions 1 year after a mild ABI. The standard physical and neurological exams were non-contributory. Crucially, baseline diffusion tensor imaging (DTI) tractography revealed rightward asymmetries in the uncinate and superior longitudinal fasciculi, suggesting reduced functional efficiency in left-lateralized verbal-affective integration. This finding provided the necessary neurofunctional indicator to apply the principle of functional compatibility in subsequent psychotherapeutic planning.

The present report describes a 1-year psychotherapeutic process in which DTI tractography and neuropsychological assessment were integrated to guide individualized treatment planning. The aim is not to evaluate treatment efficacy but to illustrate how neurofunctional information can inform clinical decision-making and support a neuroinformed approach to psychotherapy based on the principle of functional compatibility.

## 2 Case description

This single-case study was selected for its illustrative value in integrating neurofunctional data into psychotherapeutic planning. The study was conducted at the University Hospital of Gran Canaria Dr. Negrín between August 2023 and August 2024, with approval from the institutional Ethics Committee for Research with Medicines (CEIm de Las Palmas) on 29 April 2022 (protocol code: 2022-206-1). All procedures adhered to the Declaration of Helsinki, and the patient provided written informed consent, and all ethical standards were observed to ensure confidentiality and anonymity.

### 2.1 Clinical background and design

The study followed a longitudinal A–B single-case structure, including a stable baseline (A) phase before the intervention (B). This allowed for qualitative observation of change patterns while controlling for regression to the mean and external factors (Kazdin, 2021). This idiographic single-case approach follows the renewed emphasis on the methodological value of individualized designs in cognitive neurorehabilitation (Bordón Guerra et al., 2025).

The patient was an adult who had sustained a mild acquired brain injury (ABI) approximately 1 year prior to beginning treatment. The study follows an exploratory, idiographic, qualitatively oriented single-case design. The psychotherapeutic process extended over 1 year, encompassing approximately 40 sessions. Clinical information was organized into three phases: (Cuijpers et al., 2019) baseline assessment, including neuropsychological tests and diffusion tensor imaging (DTI) tractography; (Cammisuli and Castelnuovo, 2023) intervention planning, guided by interhemispheric asymmetries and functional viability; and (Wampold and Imel, 2015) post-treatment reassessment, integrating psychometric data and qualitative clinical review. The therapeutic work was conducted in an outpatient neurorehabilitation setting under continuous clinical supervision. The overall clinical timeline of the case is summarized in Table 1.

**TABLE 1:** Clinical timeline of the case.

Time point	Clinical events
**Post-injury period (pre-treatment)**	Mild acquired brain injury followed by persistent emotional dysregulation, attentional instability, and interpersonal difficulties after resolution of acute neurological findings.
**Baseline assessment (study start)**	Comprehensive neuropsychological assessment and diffusion tensor imaging (DTI) tractography conducted prior to the initiation of psychotherapeutic intervention, identifying rightward asymmetries in the uncinate fasciculus and superior longitudinal fasciculus.
**Treatment planning**	Individualized planning guided by the functional compatibility principle, matching therapeutic demands to preserved neural pathways.
**Psychotherapeutic intervention (approximately 1 year; ∼40 sessions)**	Phase-based process using neuro-oriented techniques: (1) Stabilization: sensory grounding and body-focused mindfulness to leverage right-hemisphere interoception; (2) Processing: use of visual-experiential ACT metaphors (e.g., “Passenger on the Bus”) to bypass left-hemisphere verbal load; (3) Integration: consolidation of an injury-narrative using visual timelines.
**Post-treatment assessment**	Re-evaluation of neuropsychological and emotional functioning following completion of the psychotherapeutic process; post-treatment DTI data were not collected.

Genetic factors and relevant family medical history were not considered variables of interest in this case, given the clinical focus on neurofunctional and psychotherapeutic processes.

### 2.2 Clinical and neuropsychological assessment

Emotional functioning was assessed using the Hospital Anxiety and Depression Scale (HADS). The neuropsychological battery included the Montreal Cognitive Assessment (MoCA), WAIS-III Digit Span (forward and backward), Trail Making Test (TMT-A and TMT-B), and the Rey–Osterrieth Complex Figure (copy), applying Spanish normative data where appropriate (Peña-Casanova et al., 2009).

The patient initially showed adequate general cognitive performance but reported fatigue, attentional instability, and difficulties in emotional regulation and social interactions. Psychologically, there was a tendency toward hypervigilance, affective overcontrol, and episodic dissociation under stress.

### 2.3 Neuroimaging assessment

Structural MRI and diffusion tensor imaging were performed on a *Philips Achieva 1.5T* scanner. Tractography reconstruction used *Elements Fibertracking* (Brainlab, v2.0), providing fractional anisotropy and fiber volume values for associative and commissural tracts (superior/inferior fronto-occipital fasciculi, superior/inferior longitudinal fasciculi, uncinate, cingulum, and corpus callosum).

Acquisition parameters were as follows: 128 × 128 matrix, 2 mm slice thickness, *b* = 1000 s/mm^2^. The tractography was interpreted by a neuroradiologist experienced in DTI. Interhemispheric asymmetry was used as a descriptive index of relative connectivity, defined as:


$$I\,A\,I\,\frac{\left(R-L\right)}{\left(R+L\right)/2}$$

where *R* and *L* represent right and left tract volumes, respectively. Asymmetries with | IAI| ≥ 0.20 were considered clinically relevant, based on structural lateralization research, the 0.20 cutoff was selected as a conservative threshold representing asymmetries exceeding typical interindividual variability (Forkel et al., 2022; Karolis et al., 2020). The evaluation of contralateral hemisphere integrity and interhemispheric balance has been highlighted as a critical factor in understanding clinical manifestations and recovery potential across various neurological conditions (Gul et al., 2024). Recent advances in personalized connectomics also highlight the importance of considering individual variability in white matter anatomy (Nozais et al., 2023).

**FIGURE 1:**
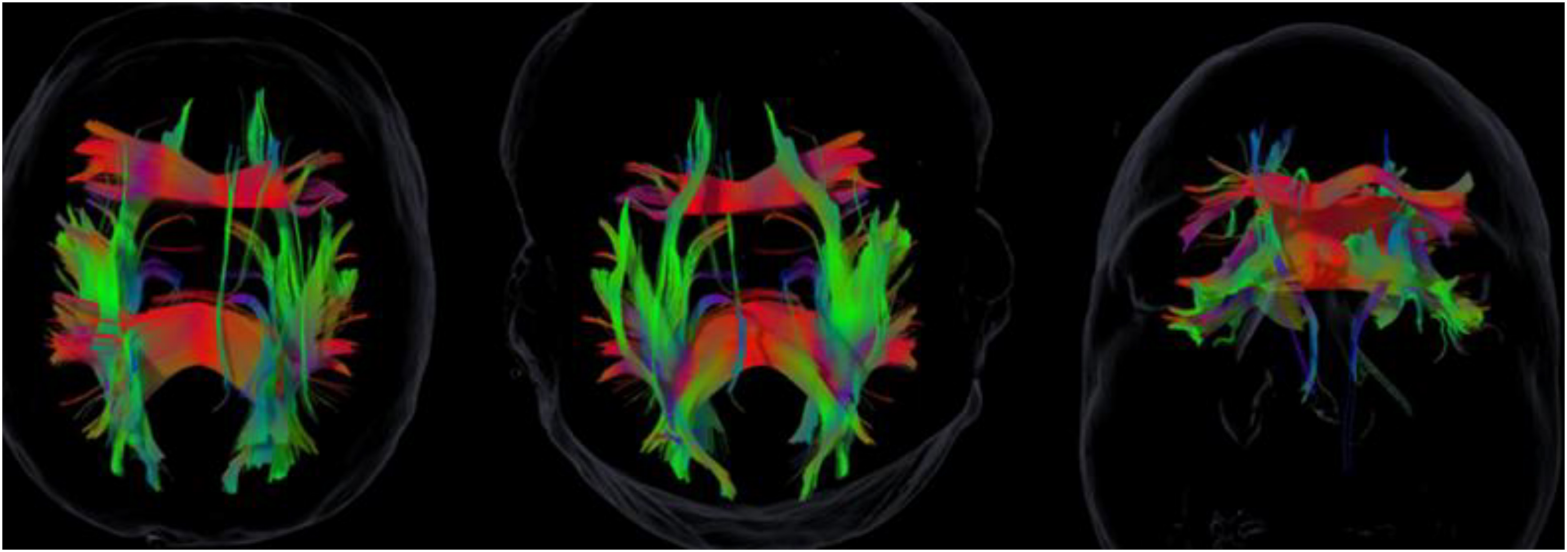
Baseline diffusion tensor imaging (DTI) tractography showing rightward asymmetries in the uncinate fasciculus (UF) and superior longitudinal fasciculus (SLF). Fiber tracking was reconstructed using Brainlab Elements (v2.0). The color map represents the principal diffusion directions: red = left-right, green = anterior-posterior, blue = inferior-superior. Note the prominence of the right UF and SLF tracts (green/red projections) reflecting the measured asymmetry.

At baseline, tractography showed globally preserved white-matter organization but with rightward asymmetries in the uncinate and superior longitudinal fasciculi (IAI ≈ +0.22 and +0.27; see Figure 1). This pattern suggested reduced efficiency of left-lateralized verbal–affective integration and greater reliance on right-hemisphere experiential and regulatory networks.

### 2.4 Therapeutic planning and procedure

#### 2.4.1 General therapeutic rationale

The psychotherapeutic approach integrated cognitive, experiential, and regulatory components, progressively adapted to the patient’s functional profile. Based on the neurofunctional findings, interventions prioritized attentional focusing, experiential awareness, and physiological regulation over highly verbal or abstract analytical work.

Sessions followed a phased structure–stabilization, guided elaboration, and consolidation–with continuous adjustment to avoid cognitive or emotional overload. Indicators of attentional saturation, dissociation, or loss of self-regulation were used as markers for pacing and adjusting techniques.

Throughout the process, special attention was given to experiential grounding and interoceptive awareness, aiming to strengthen right-hemisphere integrative mechanisms while minimizing the demand on vulnerable left-hemisphere verbal–analytic processes.

#### 2.4.2 Therapeutic techniques and clinical progression

The psychotherapeutic intervention was organized as a flexible, integrative process adapted to the patient’s evolving functional availability rather than following a fixed manualized protocol. Treatment unfolded over approximately 1 year with regular outpatient sessions and was structured into three progressive stages, each defined by the patient’s clinical stability, attentional capacity, and emotional tolerance.

During the initial stage, the primary focus was emotional regulation and stabilization. Interventions aimed to reduce physiological hyperactivation and attentional fragmentation through basic grounding strategies, including body-focused mindfulness exercises, paced breathing, and the establishment of daily routines related to sleep, activity, and self-care. Psychoeducational elements were introduced at a concrete and experiential level, deliberately avoiding complex symbolic or highly abstract material that could exceed the patient’s regulatory capacity at that stage.

In the intermediate stage, therapeutic work expanded to relational and emotional processing. Sessions addressed the patient’s avoidance of emotional engagement and anxiety related to interpersonal dependency, with particular attention to sustaining affective contact without withdrawal or overload. Selected sessions included the participation of the patient’s partner, facilitating the validation of relational changes following the injury and supporting the reorganization of roles within the couple. This phase was characterized by a gradual increase in emotional tolerance and a reduction in anticipatory anxiety.

The final stage focused on integrative and narrative work. Once sufficient regulatory stability had been established, therapy facilitated the construction of a coherent and emotionally tolerable narrative of the injury and its consequences. This process supported the redefinition of the patient’s sense of self and reduced the persistence of threat-based interpretations associated with the brain injury. At this stage, the patient demonstrated greater reflective capacity, sustained engagement, and increased flexibility in emotional processing.

Across all stages, therapeutic pacing and technique selection were continuously adjusted based on clinical indicators such as attentional fatigue, emotional saturation, or signs of dysregulation. The intervention emphasized working within preserved functional capacities while avoiding excessive cognitive or emotional load, allowing therapeutic change to emerge progressively through functional optimization rather than forced analytic elaboration.

In this case, techniques derived from ACT and mindfulness were not implemented as standardized therapeutic protocols, but were selectively employed as functionally adaptive tools aligned with the patient’s preserved neurofunctional capacities, thereby operationalizing the functional compatibility framework described in this study. To operationalize the functional compatibility principle, mindfulness-based interventions focused on body scanning and interoceptive awareness, leveraging the preserved integrity of the right uncinate fasciculus for emotional regulation. Instead of traditional Socratic questioning–which imposes a high load on left-hemisphere verbal-analytic systems–we utilized visual metaphors from Acceptance and Commitment Therapy (ACT), such as the “Passenger on the Bus” metaphor. These were presented via visual aids and experiential exercises to facilitate cognitive defusion while minimizing linguistic processing demands. Attentional training emphasized “grounding” in immediate environmental stimuli, avoiding multi-step verbal instructions that could saturate the patient’s limited working memory capacity.

### 2.5 Functional compatibility principle in practice

Given the rightward asymmetry in the uncinate and superior longitudinal fasciculi, interventions emphasized right-hemisphere experiential processes. Techniques derived from Acceptance and Commitment Therapy (ACT) and mindfulness approaches (Farb et al., 2013; Hayes et al., 2012) enhanced interoceptive awareness and emotional regulation, minimizing verbal–analytic load on left-hemisphere systems.

### 2.6 Post-treatment assessment

After 1 year of psychotherapy, re-evaluation indicated improvements across multiple domains: attention, working memory, cognitive flexibility, and emotional regulation. Anxiety decreased to non-clinical levels, accompanied by an adaptive increase in affective awareness. Table 1 summarizes the neuropsychological and emotional results before and after treatment.

Clinically, the patient transitioned from reactive and fragmented coping to a more regulated, self-reflective functioning. Impulsivity and physiological hyperreactivity diminished, while tolerance for affect and the capacity for self-observation increased.

No post-treatment DTI was performed, as the primary aim was to evaluate functional reorganization inferred from behavioral and psychometric changes, rather than structural modification (Goya-Maldonado et al., 2025; Menon, 2023; Perez et al., 2025).

## 3 Results

After 1 year of psychotherapy (approximately 40 sessions), reassessment indicated improvements across cognitive, emotional, and interpersonal domains, consistent with functional optimization within structurally preserved networks.

### 3.1 Neuropsychological and emotional results

Table 2 summarizes the pre- and post-treatment measures. Performance improved in attention, cognitive flexibility, and processing speed. The patient showed faster completion times on the Trail Making Test (A: 62 → 45 s; B: 190 → 128 s), greater balance between immediate and working memory in the WAIS-III Digit Span (forward/backward = 9/4 → 8/6), and an increase in MoCA score (25 → 28). Copy performance on the Rey–Osterrieth Complex Figure rose from the 50th to the 60th percentile, indicating better planning and visuospatial control.

**TABLE 2:** Neuropsychological and emotional results before and after intervention.

Measure	Pre	Post	Interpretation
MoCA (0–30)	25	28	Improved attention and flexibility; score moved above the cognitive impairment cut-off.
Digits WAIS-III (forward/backward)	9/4	8/6	Balanced immediate vs. working memory; improved sustained attention.
TMT-A (s)	62	45	Faster processing speed
TMT-B (s)	190	128	Improved cognitive flexibility (32% reduction, exceeding the 25% MCID threshold).
Rey Complex Figure (copy, percentile)	50th	60th	Slight improvement in planning/visuospatial control
HADS-A	11	6	Anxiety reduced to non-clinical range (Reliable Change Index, RCI = −2.1).
HADS-D	6	8	Adaptive increase in affective awareness (expected clinical progression).
TECA – Understanding/Perspective-taking	36/34	42/40	Improved mentalization and cognitive empathy
Subjective well-being (1–5)	3	4	Greater emotional stability and clarity

Emotionally, anxiety scores on the HADS decreased from 11 to 6, shifting from a clinical to a non-clinical range, while depressive symptoms slightly increased (6 → 8), reflecting adaptive affective awareness rather than psychopathology. This change was statistically reliable according to the Reliable Change Index (RCI = −2.1; Jacobson and Truax, 1991) and is generally considered clinically meaningful in applied neuropsychological and psychotherapeutic contexts, particularly within single-case designs. Empathy measures (TECA – Understanding/Perspective-taking) rose from 36/34 to 42/40, suggesting improved mentalization and cognitive empathy. The patient’s subjective well-being rating increased from 3 to 4 (on a 1–5 scale), indicating enhanced emotional stability and clarity.

The observed improvements met established criteria for Minimal Clinically Important Differences (MCID) in several domains^555^. Specifically, the reduction in TMT-B completion time (32% improvement) significantly exceeds the 20%–25% threshold typically considered representative of genuine functional gain in brain injury populations, rather than mere practice effects^66666^. Furthermore, the decrease in the HADS-A score (11 to 6) represents a critical clinical transition, as the patient moved from the “clinical case” range to the “non-clinical” range, falling below the standard cut-off of 8 points^7777^. The Reliable Change Index ($RCI = −2.1$) further confirms that these emotional changes are statistically robust and not attributable to measurement error.

### 3.2 Clinical and behavioral change

Clinically, the patient evolved from reactive, fragmented coping to a more regulated and reflective style of self-management. Impulsivity and physiological hyperreactivity decreased, while tolerance of affect and sustained emotional ambivalence improved. The patient reported greater capacity to recognize and verbalize emotions without dissociation or avoidance.

Qualitatively, therapy facilitated the integration of bodily, emotional, and cognitive signals through attentional and experiential work. The observed functional improvements were consistent with compensatory engagement of right-hemisphere networks–particularly those implicated in experiential regulation and self-awareness (Bubb et al., 2018; Forkel et al., 2022).

### 3.3 Neurofunctional interpretation

No post-treatment DTI was obtained, as the study aimed to evaluate functional reorganization inferred from behavioral and psychometric change, rather than structural modification (Goya-Maldonado et al., 2025; Menon, 2023; Perez et al., 2025). The pattern of clinical recovery–reduced anxiety, enhanced cognitive flexibility, and increased affective awareness–suggests that therapeutic change may occur through optimization of pre-existing neurofunctional resources rather than through measurable anatomical plasticity.

Together, these findings support the hypothesis that psychotherapeutic effectiveness can emerge when the technical demands of treatment are functionally compatible with the patient’s available neurocognitive capacities.

## 4 Discussion

The integration of neurofunctional indicators into psychotherapeutic planning provided actionable guidance in this mild Acquired Brain Injury (ABI) case, supporting stable clinical change consistent with functional reorganization in anatomically preserved networks. The findings suggest that therapeutic effectiveness can emerge not only from the quality of the therapeutic alliance or the chosen model (Cuijpers et al., 2019; Wampold and Imel, 2015; Zilcha-Mano, 2021), but also from the degree of alignment between the intervention’s cognitive–emotional demands and the patient’s available neurofunctional capacities. This principle–functional compatibility–frames change as an emergent property of interventions that operate within viable neural resources rather than against compromised systems. The observed pattern of recovery–reduced anxiety (HADS-A), increased cognitive flexibility, and enhanced affective awareness–corresponds to the reinforcement of right-hemisphere and interhemispheric networks associated with experiential regulation and emotional integration (Bubb et al., 2018; Forkel et al., 2022). The interpretation of the slight increase in HADS-D (6 → 8) as an adaptive rise in affective awareness rather than psychopathology aligns with the literature on experiential and mindfulness-based therapies, where a temporary amplification of emotional sensitivity often precedes regulatory integration (Elliott et al., 2013; Hayes et al., 2012). This outcome is consistent with recent evidence that psychotherapy fosters network-level neuroplasticity without requiring measurable structural remodeling (Goya-Maldonado et al., 2025; Menon, 2023; Perez et al., 2025).

In the present report, neuroplasticity is conceptualized primarily in functional rather than strictly structural terms. While a substantial body of research operationalizes neuroplasticity as measurable longitudinal change in brain structure or connectivity, clinical recovery may also involve adaptive reweighting, optimization, or inhibition of existing networks without detectable structural modification. This distinction is particularly relevant in psychotherapeutic contexts, where change is predominantly expressed at experiential, behavioral, and regulatory levels, and where neuroimaging findings are used to inform clinical decision-making rather than to serve as outcome markers of structural change (Cramer et al., 2011).

The clinical application in this case highlights a structured Neuroinformed Decision Process:

- Neurofunctional Identification: determining viable and vulnerable networks (e.g., left-hemisphere verbal-analytic tracts) via DTI and neuropsychology.
- Functional Matching: selecting therapeutic techniques (e.g., experiential and attentional methods) that are functionally compatible with preserved systems.
- Load Management: calibrating the pace and emotional load of the intervention to prevent cognitive overload and dissociative responses.

This integration promotes therapeutic safety, coherence, and efficiency. The finding of rightward asymmetries in the uncinate fasciculus (UF) and superior longitudinal fasciculus (SLF) specifically guided the prioritization of experiential and grounding techniques (Acceptance and Commitment Therapy, mindfulness), minimizing the verbal-analytic load on the vulnerable left-hemisphere system. This perspective invites reflection on therapeutic change as a progressive reorganization of functional cooperation among distributed networks, optimizing neural synchrony and efficiency within intact structures (Goya-Maldonado et al., 2025; Menon, 2023).

Beyond its illustrative clinical value, this case suggests that neurofunctional compatibility may constitute a relevant explanatory variable for understanding variability in psychotherapeutic response. Rather than proposing a specific intervention model, the present report highlights how alignment–or misalignment–between the cognitive–emotional demands of psychotherapy and the patient’s available neurofunctional resources may influence therapeutic effectiveness. From this perspective, functional compatibility may be considered a conceptual dimension for interpreting why certain psychotherapeutic approaches succeed or fail, both in individuals with acquired brain injury and, more broadly, across clinical contexts.

### 4.1 Patient perspective

From the patient’s perspective, therapy facilitated a gradual reduction in anxiety and an increased capacity to tolerate and recognize emotional experiences without avoidance or dissociation. The patient reported greater emotional clarity, improved interpersonal functioning, and a renewed sense of coherence in daily life following the injury.

### 4.2 Limitations and future directions

Despite its clinical and conceptual value, this case report has inherent limitations. First, as an exploratory, single-case study, generalizability is intrinsically low, and causal inferences must be interpreted cautiously. While the longitudinal A–B design helps control for regression to the mean, time and non-specific factors cannot be entirely ruled out (Kazdin, 2021). Critically, although practice effects are a potential confound in longitudinal assessments, the 1-year interval between evaluations and the 32% magnitude of change in high-complexity tasks–such as the TMT-B–suggest that these gains reflect a meaningful optimization of neural resources rather than test-retest familiarity. Second, the absence of post-treatment DTI data is a methodological constraint. Although post-treatment DTI data would have strengthened the neurobiological interpretation, the primary aim of this case report was not to assess structural change, but to illustrate how baseline neurofunctional information can guide clinically meaningful psychotherapeutic decision-making. While we infer functional reorganization from psychometric and behavioral change, structural validation requires future studies featuring longitudinal imaging and functional connectivity analyses (Goya-Maldonado et al., 2025; Menon, 2023).

Furthermore, the asymmetry threshold of | IAI| ≥ 0.20 is a descriptive index that requires validation in larger clinical cohorts to establish its precise prognostic relevance. Despite these methodological constraints, context-sensitive case analyses like this are increasingly recognized as essential to neuropsychological recovery research (Allum et al., 2025). Future investigations should include randomized controlled trials to formally validate the functional compatibility construct and comparative studies to assess whether tailored interventions yield superior outcomes than standard protocols, thereby advancing the field of precision psychotherapy (McCracken, 2023; Norcross and Lambert, 2019).

## 5 Conclusion

This case highlights the clinical value of integrating neurofunctional data into psychotherapeutic planning. By aligning the technical demands of psychotherapy with the patient’s viable neurocognitive resources, meaningful psychological change can emerge through functional optimization without requiring measurable structural reorganization (Goya-Maldonado et al., 2025; Perez et al., 2025). The concept of functional compatibility offers a practical and theoretically grounded framework for neuroinformed psychotherapy, emphasizing that treatment effectiveness depends on the fit between therapeutic strategies and the brain’s current functional capacity (Forkel et al., 2022; Menon, 2023). This integrative perspective bridges neuroscience and clinical practice, supporting a precision-based approach to psychological care (Lutz et al., 2021; Norcross and Lambert, 2019).

## Data Availability

The original contributions presented in this study are included in this article/supplementary material, further inquiries can be directed to the corresponding author.
